# Neuroprotective Effects of a New Derivative of Chlojaponilactone B against Oxidative Damaged Induced by Hydrogen Peroxide in PC12 Cells

**DOI:** 10.3390/molecules27186049

**Published:** 2022-09-16

**Authors:** Shaoxia Ye, Qiyin Wen, Longping Zhu, Chunguo Qian, Depo Yang, Zhimin Zhao

**Affiliations:** 1 School of Pharmaceutical Sciences, Sun Yat-sen University, Guangzhou 510006, China; 2 The Second Affiliated Hospital of Xiamen Medical College, Xiamen 361021, China

**Keywords:** sesquiterpene, neuroprotection, PC12 cells, ROS, Nrf2

## Abstract

A new sesquiterpenoid (**1**) was obtained by hydrogenating Chlojaponilactone B. The structure of **1** was elucidated according to a combination of NMR, HRESIMS, and NOE diffraction data. The treatment of H_2_O_2_ in a PC12 cell model was used to evaluate the antioxidant activity of **1**. An MMT assay showed that **1** had no cytotoxicity to the PC12 cell and rescued cell viability from the oxidative damage caused by H_2_O_2_. The treatment of **1** stabilized the mitochondria membrane potential (MMP), which decreased the intracellular ROS level and reduced cell apoptosis in the oxidative stress model. The activities of antioxidant enzyme superoxide dismutase (SOD) and glutathione peroxidase (GSH-Px) and the content of intracellular glutathione (GSH) were significantly enhanced after the treatment of **1**. In addition, the results of qRT-PCR showed that **1** treatment minimized the cell injury by H_2_O_2_ via the up-regulation of the expression of nuclear factor erythroid 2 (Nrf2) and its downstream enzymes Heme oxygenase 1 (HO-1), glutamate cysteine ligase-modifier subunit (GCLm), and NAD(P)H quinone dehydrogenase 1 (Nqo1). Based on the antioxidant activity of **1**, we speculated its potential as a therapeutic agent for some diseases induced by oxidative damage.

## 1. Introduction

Oxidative stress refers to a reaction in which the body generates a large number of reactive oxygen species after being stimulated by harmful stimuli, leading to an imbalance between the oxidation state and antioxidant state, resulting in pathological changes in tissues and cells [1]. Excessive amounts of highly active molecules are produced when the body is subjected to various stimuli, such as reactive oxygen species (ROS), causing the balance of oxidative and antioxidant states to be lost, which, in turn, results in tissue oxidative damage. Excessive free radicals produced by oxidative stress can directly or indirectly damage DNA, oxidize proteins, and reduce biological activity because of structural and functional defects [2]. Studies have shown that oxidative stress is closely related to neurological diseases, including Parkinson’s disease and Alzheimer’s disease [3,4], cardiovascular diseases and prostatic diseases [5], inflammation, and cancers [6]. The reduction in oxidative stress-induced damage to the body has become key in treating these clinical diseases. Treatment strategies based on the antioxidant amelioration of ROS appear to be able to delay the progression of these diseases.

Natural products are rich in resources and diverse in structure. Numerous studies have shown that antioxidants from natural plant sources have neuroprotective effects to reduce the probability of human diseases [7,8,9]. Demethylenetrahydroberberine was reported to protect dopaminergic neurons and alleviates the behavioral disorder in a mouse model of Parkinson’s disease through anti-apoptotic, anti-inflammatory and antioxidant effects [10]. Chen et al. found that GTS40, which is an active fraction of Gou Teng-San, helped prevent and treat oxidative stress-mediated neurodegenerative disorders [11]. Antioxidant peptides from protein hydrolysate of skipjack tuna milt showed protection for human umbilical vein endothelial cells under H_2_O_2_ stimuli [12]. A combination of natural antioxidants (Vitamin E, quercetin, and basil oil) is potential innovation against Alzheimer’s disorder [13]. Therefore, research on new natural compounds with neuroprotective activity are highly urgently and necessary. Finding lead compounds with novel structures and significant activity from natural products has become an effective way to develop new drugs. However, traditional compounds obtained by separation and extraction are limited in yield and cannot satisfy the needs of research and development. Chemical modifications or biotransformation of compounds have become important methods to improve pharmacological activity, reduce side effects, and increase drug stability.

The plants of genus *Chloranthus* are widely applied for Traditional Chinese Medicine to treat bruises, rheumatic arthralgia, pain, soreness, and furunculosis [14]. Many terpenoids have been reported, such as terpenoids, diterpenoids, sesquiterpenoid dimers, and sesquiterpene lactones, in phytochemical investigations of *Chloranthus* plants. Pharmacological research has shown that *Chloranthus* plant have anti-inflammatory, anti-tumor, antivirus, and antifungal activities [15]. Sesquiterpenoids are a diverse group of compounds with abundant pharmacological activity, which have attracted the attention of scholars in recent years. By modifying the structure of sesquiterpenoids with lower toxicity and higher activity, they can be used in new drugs, food and cosmetics. Our previous studies showed that chlojaponilactone B, a sesquiterpenoid isolated from the genus *Chloranthus*, has anti-inflammatory effects that rely on the C-6 acetyl group and the C-8–C-9 double bond [16,17]. In this study, we perhydrogenated chlojaponilactone B to explore its structure–activity relationship. The sesquiterpene compound chlojaponilactone B was modified to reduce the three double bonds in the structure and open the ring of cyclopropane to obtain a derivative, termed compound, **1**. Surprisingly, we obtained a new compound (**1**) with strong anti-oxidant activities and inhibition of nitric oxide (NO) production (the value of IC_50_ is shown in Supplementary Materials). The aim of this study was to investigate the neuroprotective effect of compound **1** against oxidative damage to PC12 cells induced by H_2_O_2_.

## 2. Results and Discussion

### 2.1. Chemical Modification and Structure Elucidation

Our previous study speculated that the anti-inflammatory effects of Chlojaponilactone B depended on the C-6 acetyl group and the C-8–C-9 double bond [17]. To test this hypothesis, we perhydrogenated chlojaponilactone B into compound **1**.

Compound **1** appeared as a white powder and has the molecular formula C_17_H_26_O_4_ according to the HRESIMS spectrum at *m/z* 317.1724 [M + Na] ^+^ (calculated as 317.1724), implying five indexes of hydrogen deficiency. ^1^H-NMR and ^13^C-NMR analysis of **1** (Table 1) revealed signals for protons representing five methyls (δ_H_ 0.80 (3H, s), 0.86 (3H, d, *J* = 6.76 Hz), 0.92 (3H, d, *J* = 7.00 Hz), 1.25 (3H, d, *J* = 7.36 Hz), and 2.02 (3H, s)). The ^13^C-NMR spectra, combined with DEPT 135 experiments, revealed seventeen signals, comprising one ester group (δ_C_ 178.4), one acetyl group (δ_C_ 169.9), five methyl groups (δ_C_ 10.4, 13.3, 16.2, 19.2, 21.8), two sp^3^ hybridized methylene groups, seven sp^3^ hybridized methine groups (two of which contained oxygens), and one sp^3^ hybridized quaternary carbon. Among the five unsaturated positions, one is occupied by ester groups, one is occupied by an acetyl group, and the remaining three unsaturated positions are presumed to be in a tricyclic structure in **1** (see Table 1).

The analysis and identification of the structure of compound **1** was achieved using various 2D NMR spectroscopic techniques. The heteronuclear multiple bond correlations (HMBCs) of H_2_-2/C-1, C-3 and C-10, H_3_-15/C-3, C-4 and C-5, H_3_-14/C-1, C-5, C-9, and C-10 indicated that C-1, C-3, C-4, C-5, and C-10 formed the five-membered ring, and the C-1, C-4, and C-10 positions were connected to one methyl group, respectively, which was supported by the ^1^H-^1^H correlated spectroscopy (COSY) correlations of H_3_-2/H-1/H_2_-3/H-4/H-5 and H-4/H_3_-15. The HMBCs of H_3_-14/C-5, C-9, and C-10, combined with the ^1^H-^1^H COSY correlations of H-5/H-6/H-7/H-8, suggested that the six-membered ring was formed by C-5, C-6, C-7, C-8, C-9, and C-10, for which a C-5–C-10 bridge connected it to the five-membered ring. The HMBC correlations of H_3_-13/C-7, C-11, and C-12, combined with the ^1^H-^1^H COSY correlations of H-7/H-11/H_3_-13, verified that C-7, C-8, C-11, and C-12 formed the furan lactone ring, for which a C-7–C-8 bridge connected it to the six-membered ring, and the C-11 position was connected to one methyl group. The HMBC correlations of H-6/COOCH_3_ indicated that the C-6 position was modified with an acetyl group. Therefore, the planar structure of **1** was identified as a 2,3-ring guaianese sesquiterpene.

**Figure 1:**
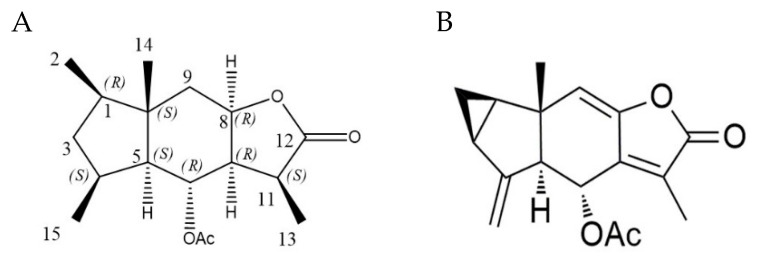
The structures of (**A**) Compound **1** (Perhydrochlojaponilactone B); (**B**) Chlojaponilactone B.

We analyzed its nuclear Overhauser effect (NOE) spectrum and compared its configuration with the known compound, chlojaponilactone B (Figure 1), which determined the absolute configuration of **1**. Compound **1** is the perhydrogenated and cyclopropyl ring-opened product of Chlojaponilactone B. After opening the cyclopropyl moiety, C-1 maintains the *R* configuration, and C-5, C-6, and C-10 keep their *S*, *R*, and *S* configurations, respectively. The NOE spectrum indicated that H-6 is related to H_3_-13/H_3_-14/H_3_-15; H_3_-14 is related to H_3_-15; and H-4 is related to H-5, which determined C-4 as being in the S configuration. The correlation between H-8 and H-7/H-11, the correlation between H-7 and H-11, and the coupling constant of H-7 and H-8 was 1.52, which suggested that H-7 and H-8 are on the same side, and H-6 and H_3_-13 in the binding spectrum are related; therefore, the absolute configurations of C-7, C-8, and C-11 were determined as *R*, *R,* and *S*, respectively. Thus, the structure of compound **1** was established as depicted in Figure 1, and was named Perhydrochlojaponilactone B.

### 2.2. Neuroprotective Effect of Compound ***1*** against PC12 Cell Injury Induced by H_2_O_2_

PC12 cells were cloned from rat adrenal pheochromocytoma and differentiated into sympathetic nerve cells using nerve growth factor (NGF) stimulation, which have been widely used in studies of neurological diseases [18]. PC12 cell treating with H_2_O_2_ is a common model to study oxidative damage, which will cause cell membrane and nuclear damage, the loss of mitochondrial membrane potential (MMP), and the decreased activities of antioxidant enzymes, containing glutathione peroxidase (GSH-Px), superoxide dismutase (SOD), and the cellular glutathione (GSH) content [19,20,21].

**Figure 2:**
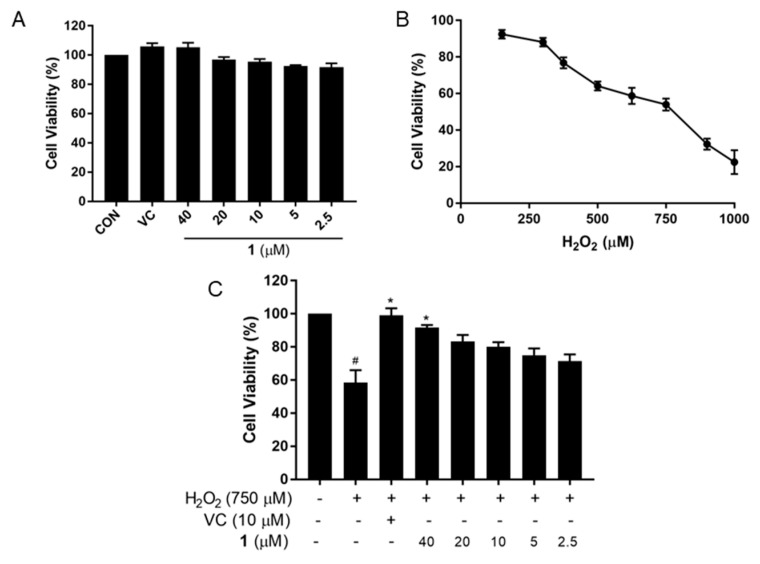
Compound **1** had no cytotoxicity to PC12 cells and ameliorated oxidative damage induced by H_2_O_2_ treatment. (**A**) Cell viability after exposed to various concentrations of **1** (2.5, 5, 10, 20 and 40 μM) or VC for 24 h; (**B**) cells were treated with different concentrations of H_2_O_2_ for 24 h; (**C**) cell viability after treatment of **1** or VC in H_2_O_2_−induced oxidative stress cell model. Cells treated with VC as the positive control. ^#^ *p* < 0.05 vs. control cells. * *p* < 0.05 vs. H_2_O_2_-treated cells.

PC12 cells were exposed to different concentrations of compound **1** to determine its cytotoxicity, as assessed using an MTT-based colorimetric test. As shown in Figure 2A, cell viability approached 100% at the concentration of 40 μM, 20 μM, 10 μM, 5 μM, and 2.5 μM, suggesting that **1** had no cytotoxicity to PC12 cell. To choose a proper concentration of H_2_O_2,_ cells were treated with varying concentrations for 24 h. With increasing H_2_O_2_ concentration, the cell viability decreased in a linear manner (53.94% at 750 μM) (Figure 2B). Therefore, we selected 750 μM H_2_O_2_ to induce oxidative damage in at least half of the viable cells. The H_2_O_2_-induced decrease in cell viability was ameliorated dramatically after treatment with **1** in a dose-dependent manner. Cell viability after treatment of **1** at 40 μM was approaching to that treated with Vitamin C (VC, 10 μM), which was dramatically enhanced compared to the group induced with H_2_O_2_ (*p* < 0.05). In particular, **1** displayed a strong anti-oxidative effect at a lower concentration (2.5 μM) (Figure 2C). These results confirmed the non-cytotoxicity and antioxidant activity of **1** on PC12 cells.

### 2.3. Effects of ***1*** on ROS Generation in H_2_O_2_-Induced PC12 Cells

**Figure 3:**
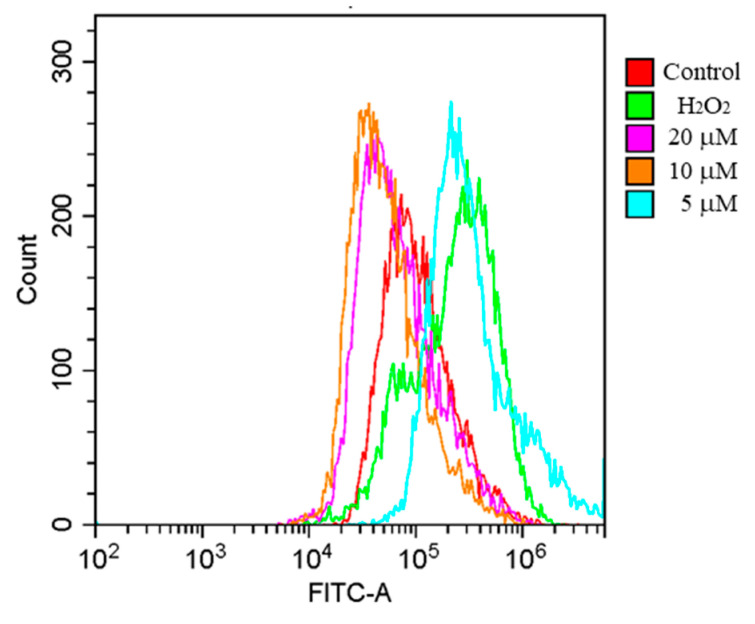
Effect of **1** on ROS production in PC12 cells induced by H_2_O_2_, as determined using flow cytometry. Cells were treated with various concentrations of **1** (5, 10, 20 μM) combined with H_2_O_2 (_750 μM); control group is treated with 0.5% dimethyl sulfoxide (DMSO) for 24 h.

Oxidative stress leads to neutrophil infiltration, and increased secretion of nucleic acids [22], ultimately caused various chronic diseases. ROS are derivatives of free radicals, and include hydrogen peroxide, singlet oxygen, and ozone. ROS in the body have certain functions, such as participating in immune and signal transduction processes. In the normal physiological state, the production and clearance of free radicals in the body maintain a dynamic balance. The production and clearance of ROS is an important marker of redox homeostasis. Under normal physiological conditions, cells eliminate the accumulated ROS by generating antioxidants [23]. When the body or immune cells (macrophages and neutrophils) are subjected to harmful stimuli, ROS clearance is reduced, causing oxidative damage and even cell death [24]. Thus, excess ROS exhibits destructive behavior. As shown in Figure 3, intracellular ROS showed a burst increase in H_2_O_2_-treated PC12 cells. However, compound **1** treatment decreased ROS levels in PC12 cells after 24 h. These findings suggested that compound **1** could effectively antagonize the ROS accumulation in PC12 cells induced by H_2_O_2_.

### 2.4. Effects of ***1*** on the Recovery of the Loss of MMP in H_2_O_2_-Induced PC12 Cells

**Figure 4:**
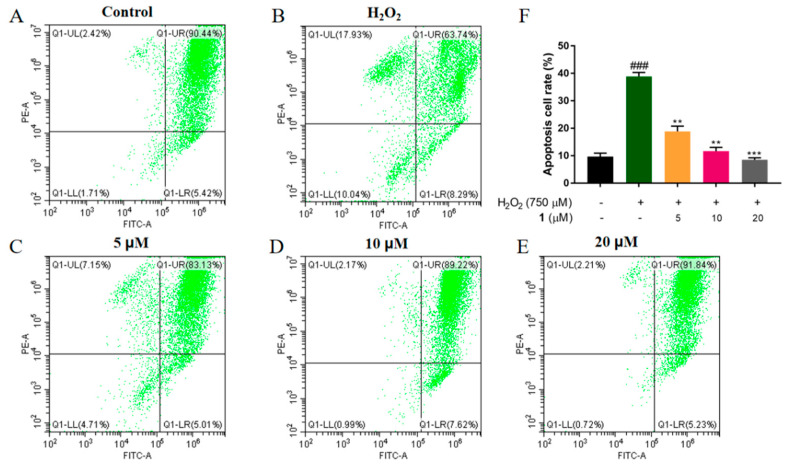
Compound **1** restored the loss of mitochondria membrane potential (MMP) in H_2_O_2_ treated PC12 cells. The MMP was assessed using flow cytometry. PC12 cells were treated with (**A**) control; (**B**) H_2_O_2_ (750 μM), and varied concentrations of compound **1** (**C**) (5 μM); (**D**) (10 μM); (**E**) (20 μM) combined with H_2_O_2_ (750 μM) for 24 h; (**F**) Apoptosis cell rate of different groups ^###^ *p* < 0.005 vs. control cells. ** *p* < 0.01, *** *p* < 0.005 vs. H_2_O_2_-treated cells.

Mitochondria are important in many biological processes, including ROS generation, apoptosis, the cell cycle, and cell growth. However, when the body is stimulated by endotoxins or alcohol, the antioxidant system is damaged, and ROS clearance is blocked. The accumulated ROS led to mitochondrial membrane damage and mitochondrial membrane potential (MMP) reduction [25]. In order to investigate H_2_O_2_-induced mitochondrial dysfunction in PC12 cells, JC-1 Kits were used for MMP detection. Moreover, apoptosis was detected quantitatively using flow cytometry. As shown in Figure 4, after exposure to H_2_O_2_, PC12 cells displayed a dramatic increase in cell apoptosis (*p* < 0.001). By contrast, **1** treatment dose-dependently decreased the number of apoptotic cells. These results suggested that **1** could restore the decrease in cellular MMP and attenuate oxidative stress-induced cell apoptosis, thus exerting a neuroprotective effect.

### 2.5. Effects of ***1*** on SOD and GSH-Px Activities, and GSH Levels in H_2_O_2_-Induced PC12 Cells

GSH-Px is an important peroxide decomposition enzyme that catalyzes GSH to generate glutathione disulfide and reduces toxic H_2_O_2_ to non-toxic hydroxyl compounds. The enzymes GSH-Px and SOD are important oxygen free radical scavengers in cells. GSH is a natural tripeptide composed of glutamate, cysteine, and glycine, comprising a sulfhydryl compound that contributes to the reductive catalysis of thiol and disulfide bonds, which plays an important role in maintaining redox homeostasis. The reduction in glutathione in the brain is associated with Parkinson’s disease and aging [26]. The antioxidant system comprising GSH, GSH-Px, and SOD maintains the body’s redox homeostasis under physiological conditions.

**Figure 5:**
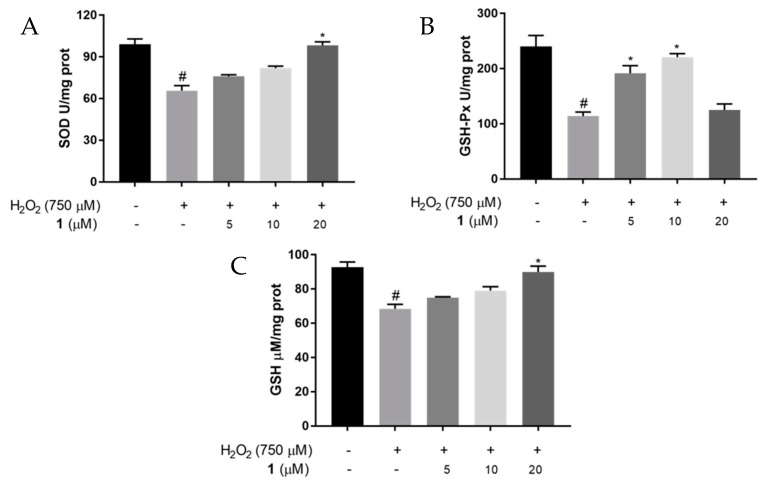
SOD, GSH−Px activities and GSH levels in PC12 cells induced with H_2_O_2_. Cells were pretreated with different concentrations of **1** (5, 10, or 20 μM) and H_2_O_2_ (750 μM) for 24 h. (**A**) The SOD; (**B**) GSH−Px activities; (**C**) the GSH level ^#^ *p* < 0.05 vs. control cells. * *p* < 0.05 vs. H_2_O_2_-treated cells.

To further explore the effect of **1** on antioxidants in H_2_O_2_-stimulated PC12 cells, we used ELISA to detect SOD and GSH-Px activities and GSH levels. The SOD activity in the H_2_O_2_ stimulation group decreased significantly compared to that in the control group (*p* < 0.05), whereas the SOD activity was enhanced after treatment with **1** in a dose-dependent manner. The GSH-Px activity decreased significantly (*p* < 0.05) in the H_2_O_2_ stimulation group, while **1** treatment enhanced the activity of GSH-Px, with the highest level at 10 μM. The GSH content in the H_2_O_2_ group decreased compared to control group (*p* < 0.05). However, the GSH content increased dose-dependently after **1** treatment (Figure 5). These findings suggested that **1** could increase antioxidant levels, thereby exerting an antioxidant effect.

### 2.6. Effects of ***1*** on the mRNA Expression Levels of Antioxidant Proteins in PC12 Cells Induced with H_2_O_2_

**Figure 6:**
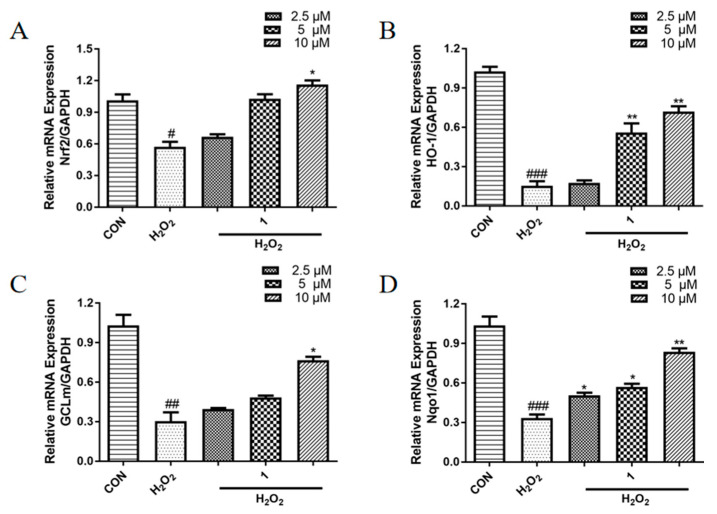
Effects of **1** on the mRNA expression levels of (**A**) Nrf2; (**B**) HO-1; (**C**) GCLm; (**D**) Nqo1. PC12 cells were incubated with compound **1** at 2.5, 5, or 10 μM, combined with H_2_O_2_ (750 μM), for 24 h before assessment using qRT-PCR. GAPDH was used as the internal control. Values are representative of three independent experiments. ^#^ *p* < 0.05, ^##^ *p* < 0.01, ^###^ *p* < 0.005 vs. control cells. * *p* < 0.05, ** *p* < 0.01 vs. H_2_O_2_-induced cells.

Furthermore, to preliminarily explore the mechanism of the protective effects of **1** against damage by H_2_O_2_ in PC12 cells, qRT-PCR was performed to detect the expression levels of nuclear factor erythroid 2 (Nrf2), glutamate cysteine ligase-modifier subunit (GCLm), heme oxygenase 1 (HO-1), and NAD(P)H quinone dehydrogenase 1 (Nqo1). Nrf2 regulates detoxification and downstream antioxidant enzyme gene expression, including Nqo1, GCLm, and HO-1. Nrf2 also regulates SOD and GSH-Px activities and the GSH level [27,28,29,30]. As shown in Figure 6, exposure to H_2_O_2_ downregulated the transcription of Nrf2, GCLm, HO-1, and Nqo1, whereas compound **1** treatment dose-dependently increased the mRNA expression levels of these antioxidant proteins. 

## 3. Materials and Methods

### 3.1. Reagents

Methyl thiazolyl tetrazolium (MTT), JC-1 Kit assay kit, 2′,7′-dichlorofluorescein diacetate (DCFH-DA) fluorescent dye were purchased from the Beyotime Institute of Biotechnology (Shanghai, China). The SOD, GSH-Px and GSH immunosorbents assay kits were purchased from Beijing Solarbio Technology (Beijing, China) Co., Ltd. All primers were purchased from Sangon Biotech (Shanghai, China) Co., Ltd. Other chemicals and solvents used in the present study were of analytical or biological grade.

### 3.2. Cell Culture

The Cell Bank of Shanghai Institute of Biochemistry and Cell Biology (Shanghai, China) provided the PC12 cells. The cells were cultured in Roswell Park Memorial Institute 1640 (RPMI 1640, Gibco, Grand Island, NY, USA) medium containing 100 U/mL penicillin (HyClone, Logan, UT, USA), 100 g/mL streptomycin (HyClone), 5% Horse Serum (Gibco), and 5% fetal bovine serum (FBS, Gibco), in a humid atmosphere containing 5% CO_2_ at 37 °C.

### 3.3. Assay of Cell Viability

A 3-(4,5-dimethylthiazol-2-yl)-2,5-diphenyltetrazolium bromide (MTT) assay was used to assess the ability of **1** to protect PC12 cells against injury induced by H_2_O_2_. PC12 cells were added to the wells of 96-well plates (5 × 10^4^ cells/mL) and cultured for 24 h. Thereafter, the cells were treated with different concentrations (40, 20, 10, 5, and 2.5 μM) of **1** or VC (10 μM) with H_2_O_2_ (750 μM). Cells treated with H_2_O_2_ (750 μM) served as the model group, those treated with 0.5% DMSO served as the blank control. Then, MTT (20 µL of a 5 mg/mL solution in sterile phosphate-buffered saline (PBS)) was added to the wells and incubated for 4 h, after which the medium was aspirated off and discarded. Finally, each well received 100 µL of DMSO, followed by microplate reader (Molecular Devices, Sunnyvale, CA, USA) detection of the absorbance (A) at 490 nm.

### 3.4. ROS Measurement

Intracellular ROS contents were determined using a 2,7-dichloro-dihydro-fluorescein diacetate (DCFH-DA) assay. PC12 cells were seeded in a 6-well plate at 1 × 10^6^ cells/mL, cultured for 24 h, and then treated with H_2_O_2_ (750 μM) as the model group. The blank group was treated with 0.5% DMSO, and the treatment group comprised cells treated with **1** at different concentrations (20, 10, and 5 μM) combined with H_2_O_2_ (750 μM) (both for 24 h). Next, 10 μM DCFH-DA was added to the cells and incubated for 30 min. Flow cytometry (Beckman coulter, Indianapolis, IN, USA) was used to observe the fluorescence of intracellular ROS.

### 3.5. Mitochondrial Membrane Potential

A JC-1 Kit was used to assess changes to the mitochondrial membrane potential, a commonly used marker of early apoptosis. PC12 cells were added to the wells of a 12-well plate at 1 × 10^5^ cells/mL and incubated for 24 h, after which they were treated with **1** at different concentrations (20, 10, and 5 μM) with H_2_O_2_ (750 μM) for another 24 h. Wells with no test compound that received only H_2_O_2_ (750 μM) served as controls; wells with neither any test compound nor H_2_O_2_ (750 μM) served as blank controls. We harvested the cells, and rinsed them using PBS, then subjected them to flow cytometry to analyze fluorescence.

### 3.6. Measurement of Intracellular Antioxidant Activity

The intracellular SOD and GSH-Px activities, and the level of GSH in PC12 cells were measured employing enzyme-linked immunosorbents assay (ELISA) kits (Solarbio, Beijing, China). Briefly, the cells were treated the same as in Section 3.5. After 24 h of stimulation, cell lysis was achieved by incubation on ice, the cell lysate was collected, and the proteins were obtained by centrifugation for 10 min at 12,000× *g* and 4 °C. A Pierce™ BCA Protein Assay Kit (Thermo Fisher, San Diego, CA, USA) was used to quantify the total proteins in the samples. The absorbance of GSH-Px, SOD, and GSH were detected at 412, 560, and 412 nm, respectively. 

### 3.7. Analysis of Antioxidant Gene Expression by Quantitative Real-Time Reverse Transcription PCR (qRT-PCR) 

PC12 cells were added to 6-well plates at 1 × 10^6^ cells/mL and incubated for 24 h. Subsequently, the model group comprised cells exposed with H_2_O_2_ (750 μM), the blank group comprised cells treated with 0. 5% DMSO, and the treatment group comprised cells treated at different concentrations of **1** (2.5, 5, and 10 μM) combined with H_2_O_2_ (750 μM) (all groups were treated for 24 h). The Trizol reagent (Invitrogen, Grand Island, NY, USA) was used to extract total RNA from the PC12 cells, and a Nanodrop 2000 ultramicro spectrophotometer (Thermo Fisher Scientific, Sacramento, CA, USA) was used to determine the RNA concentration. A HiScript II Q RT SuperMix for qPCR (Vazyme, Nanjing, China) was used to reverse-transcribe the mRNA into first strand cDNA. Next, a Hieff™qPCR SYBR^®^ Green Master Mix (Yisheng, Shanghai, China) in a LightCycler 96 Real-Time PCR System (Roche, Basle, Switzerland) was used to perform quantitative real-time PCR (qPCR) assays using the cDNA as the template. The qPCR reaction conditions comprised: preincubation at 95 °C for 5 min, then 40 cycles of denaturation at 95 °C for 10 s, annealing at 55 °C for 20 s, and elongation at 72 °C for 20 s. Table 2 shows the gene-specific oligonucleotide primers employed in qPCR. The reference gene comprised GAPDH (glyceraldehyde 3-phosphate dehydrogenase). All experiments were carried out three times.

### 3.8. Statistical Analysis

Values are presented as the means ± SD of triplicate experiments. were performed using One-way analysis variance was used to carry out the statistical analyses in SPSS 18.0 (IBM Corp., Armonk, New York, NY, USA). Statistically significant differences were accepted at a *p*-value less than 0.05.

## 4. Conclusions

In this study, the sesquiterpene compound chlojaponilactone B was modified to reduce the three double bonds in the structure and open the ring of cyclopropane to obtain a new derivative, named compound **1**. Extensive activity screening found that **1** has strong anti-oxidant activities. Compound **1** could significantly reverse the oxidative damage caused by H_2_O_2_ in the oxidative stress model of PC12 cells. Further study showed that ROS production in oxidatively damaged cells was inhibited significantly by the application of **1**. Flow cytometry showed that after H_2_O_2_ (750 μM) stimuli, ROS levels in PC12 cells increased significantly; however, after **1** intervention, ROS levels decreased clearly. Compound **1** increased the cellular MMP, and attenuate oxidative stress induced cell apoptosis. Moreover, compound **1** enhanced SOD and GSH-Px activities and GSH levels markedly; it also dose-dependently increased the mRNA expression levels of Nrf2, GCLm, HO-1, and Nqo1, proving that **1** has a strong antioxidant effect. In conclusion, Compound **1** is a potentially promising therapeutic agent to treat oxidative damage-induced diseases in future research.

## Figures and Tables

**Table 1:** ^1^H-NMR (400 MHz) and ^13^C-NMR (100 MHz) of **1** (CDCl3, δ in ppm).

Position	δ _C_	δ_H_, multi. (*J* in Hz)
1	47.0	1.48, m
2	13.3	0.86, d (6.76)
3	41.3	0.99, m 2.16, m
4	30.4	2.14, m
5	54.3	1.81, dd (12.08, 8.64)
6	70.3	5.10, dd (12.08, 8.76)
7	48.1	2.62, ddd (12.32, 5.00, 1.00)
8	80.3	4.65, td (4.56, 1.52)
9	40.7	1.30, m 2.21, m
10	43.8	
11	40.9	2.83, m
12	178.4	
13	10.4	1.25, d (7.36)
14	16.2	0.80, s
15	19.2	0.92, d (7.00)
COOCH_3_	169.9	
COOCH_3_	21.8	2.02, s

**Table 2:** Primer sequence.

Gene Symbol	Forward Primer (5’-3’)	Reverse Primer (5’-3’)
GADPH	GGTGAAGGTCGGTGTGAACG	CTCGCTCCTGGAAGATGGTG
GCLm	CTTCGCCTCCGATTGAAGATG	AAAGGCAGTCAAATCTGGTGG
HO-1	AGATGGCGTCACTTCGTCAG	GCTGATCTGGGGTTTCCCTC
Nrf2	TTGGCAGAGACATTCCCAT	GCTGCCACCGTCACTGGG
Nqo1	TGAAGAAGAGAGGATGGGAGG	GATGACTCGGAAGGATACTGAAAG

## Data Availability

The data presented in this study are available in article and Supplementary Material.

## Supplementary Materials

The following supporting information can be downloaded at: https://www.mdpi.com/article/10.3390/molecules27186049/s1, Figure S1: ^1^H-NMR spectrum of compound **1** (CDCl_3_, 400 MHz), Figure S2: ^13^C-NMR spectrum of compound **1**, Figure S3: DEPT 135°NMR spectrum of compound **1,** Figure S4: ^1^H-^1^H COSY spectrum of compound **1**, Figure S5: HSQC spectrum of compound **1**, Figure S6: HMBC spectrum of compound **1**, Figure S7: NOESY spectrum of compound **1**, Figure S8: HRESIMS of compound **1**, S9: The method of NO production, Figure S9: Dose-effect curve of compound **1** on LPS-induced NO inhibition.

## Sample Availability

Not available.
