# Supplementary material for: Neuroprotective Effects of a New Derivative of Chlojaponilactone B against Oxidative Damaged Induced by Hydrogen Peroxide in PC12 Cells
Source: Molecules. 2022 Sep 16;27(18):6049. doi: 10.3390/molecules27186049 (PMC9501937; doi:10.3390/molecules27186049)
Supplement: Supplementary file 1 [file molecules-27-06049-s001.zip › molecules-1860289-supplementary.pdf]

Supplementary Materials

Neuroprotective Effects of a New Derivative of Chlojaponilactone B against Oxidative Damaged Induced by Hydrogen Peroxide in PC12 Cells

Shaoxia Ye [1,2], Qiyin Wen [1], Longping Zhu [1], Chunguo Qian [1], Depo Yang [1] and Zhimin Zhao [1,*]

[1] School of Pharmaceutical Sciences, Sun Yat-sen University, Guangzhou 510006, China
[2] The Second Affiliated Hospital of Xiamen Medical College, Xiamen 361021, China
* Correspondence: zhaozhm2@mail.sysu.edu.cn; Tel.: +86-020-3994-3043

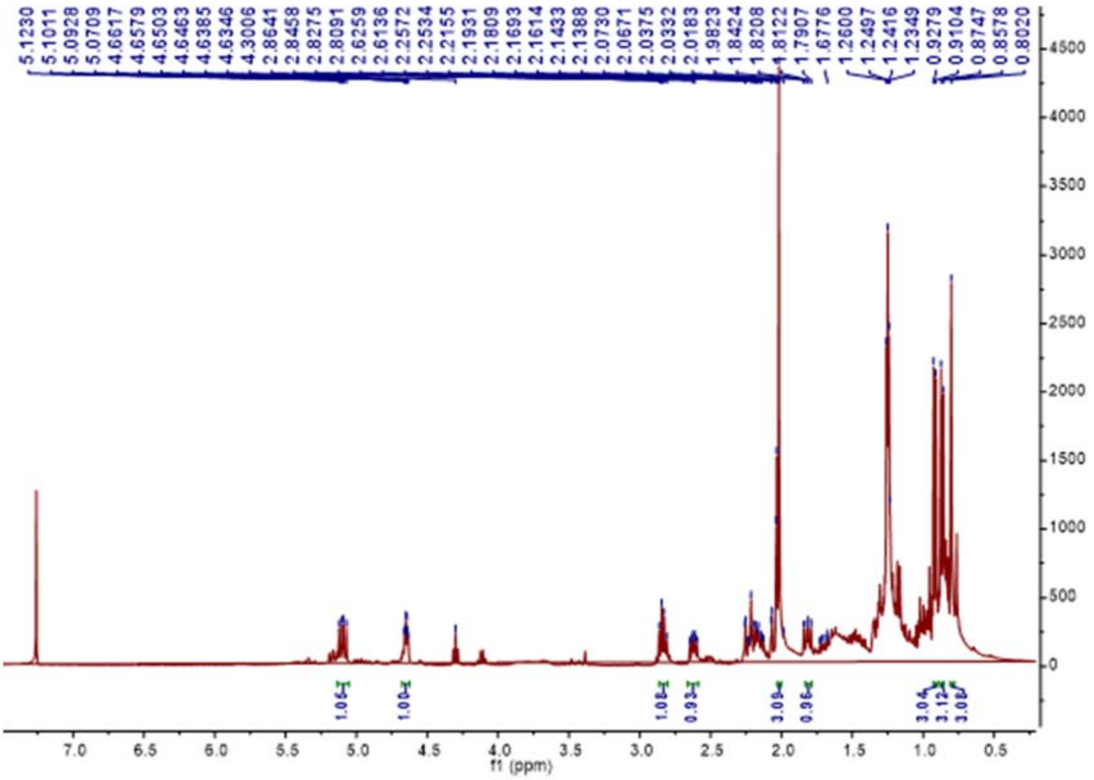

Figure S1. ^{1}H NMR spectrum of compound **1** ($CDCl_3$, 400 MHz).

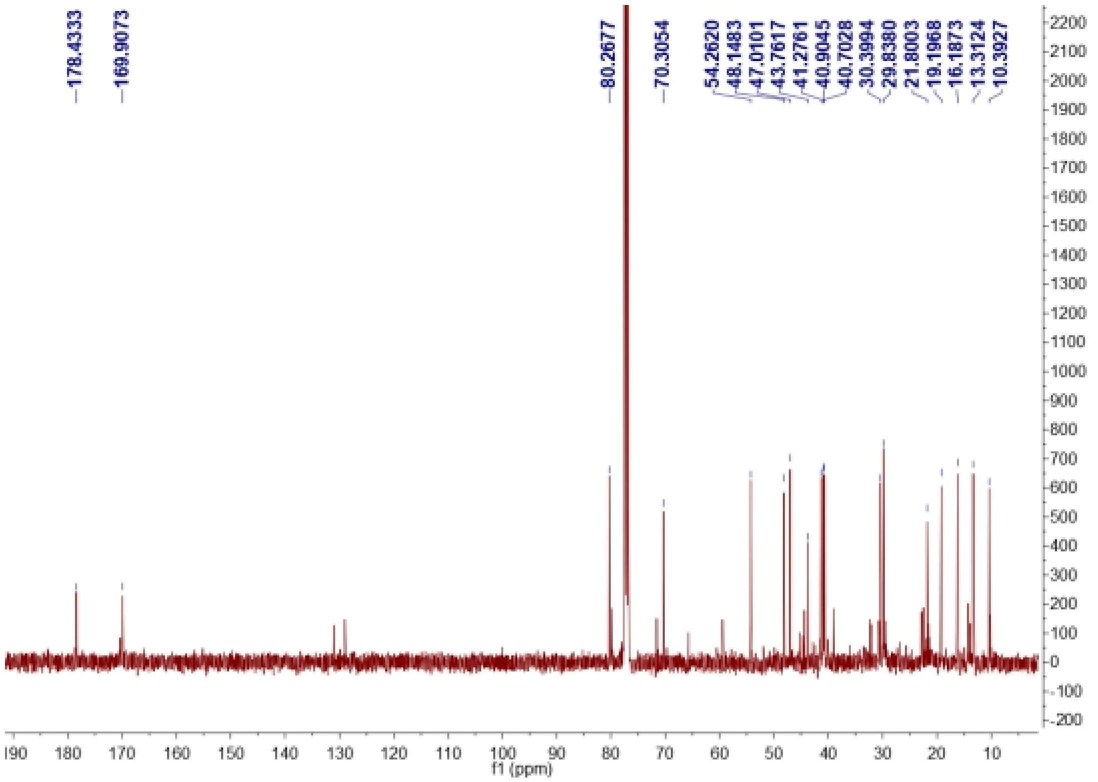

Figure S2. ^{13}C NMR spectrum of compound **1** ($CDCl_3$, 100 MHz).

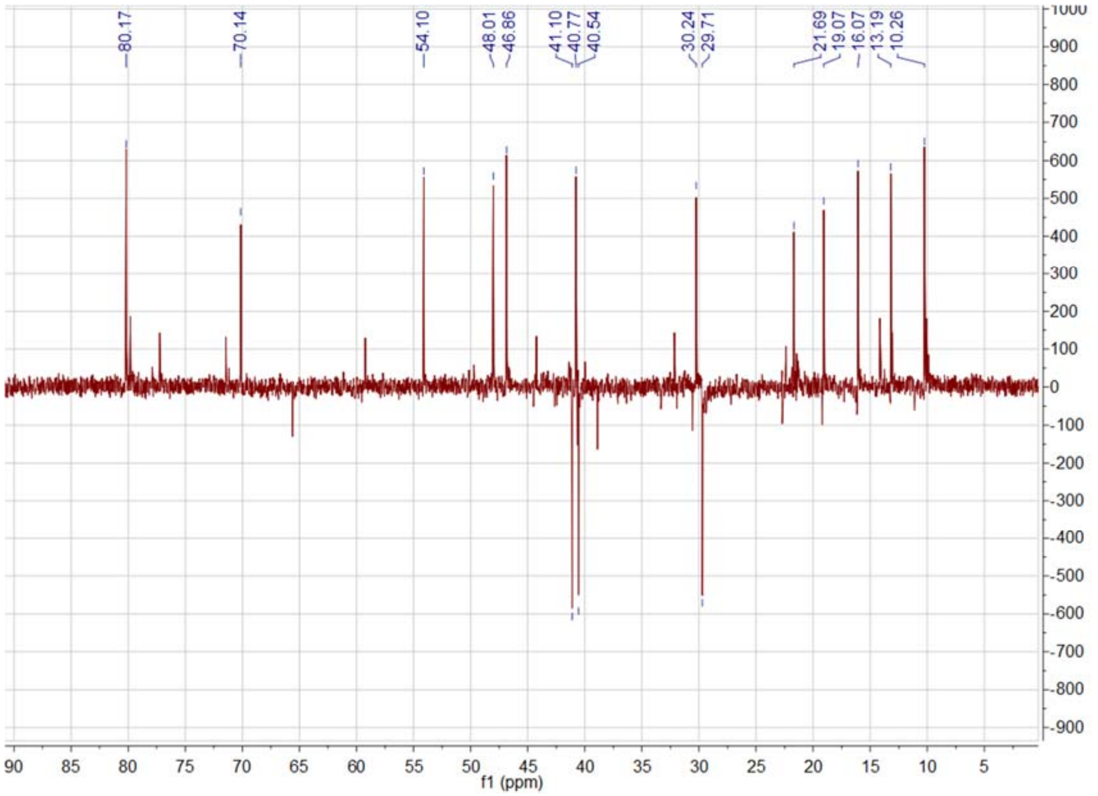

Figure S3. DEPT 135°NMR spectrum of compound **1.**

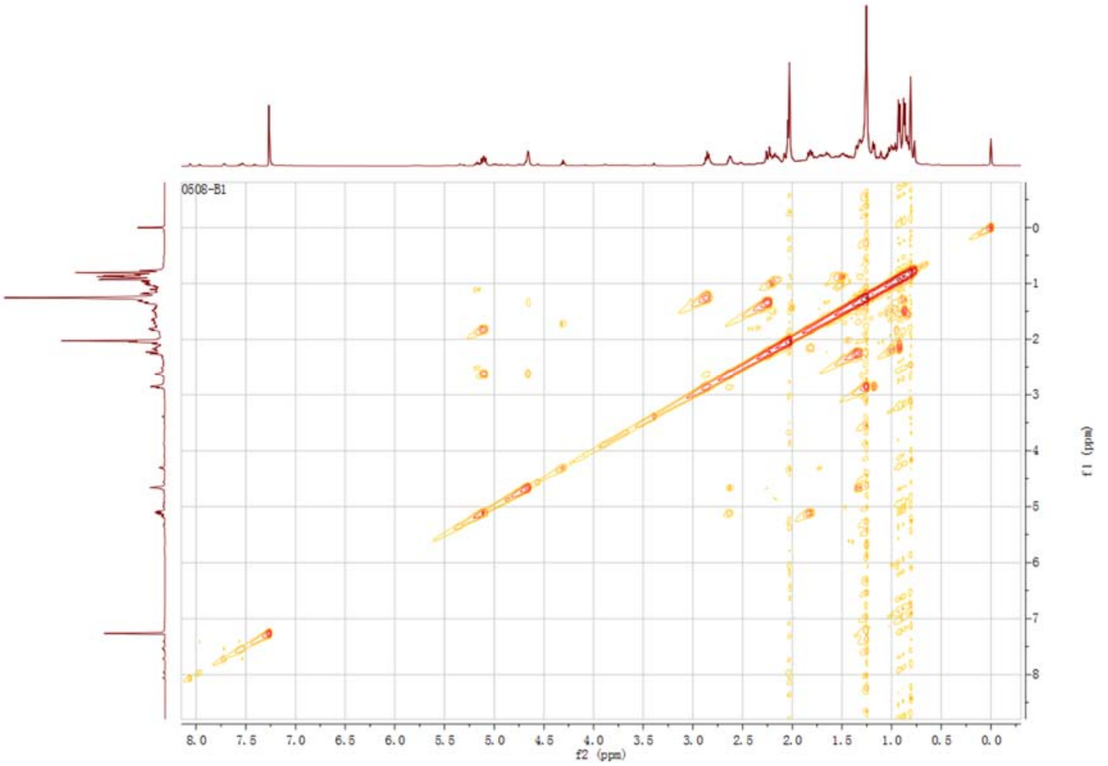

Figure S4. ^{1}H-^{1}H COSY spectrum of compound **1.**

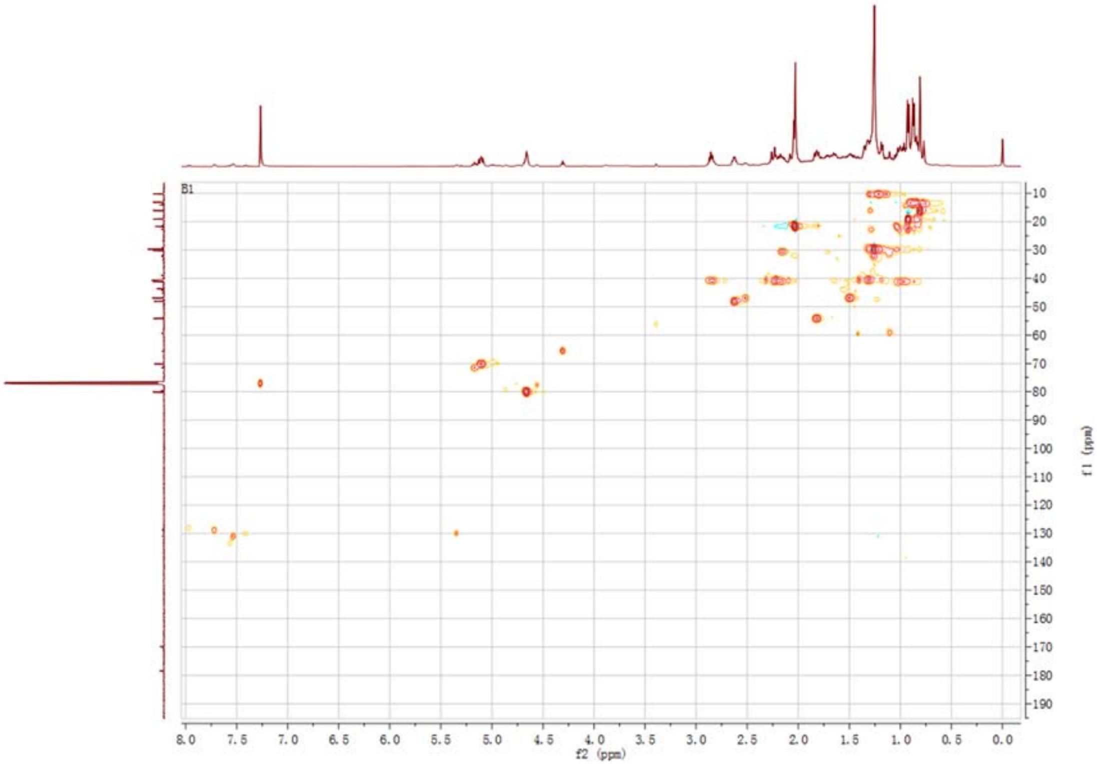

Figure S5. HSQC spectrum of compound **1.**

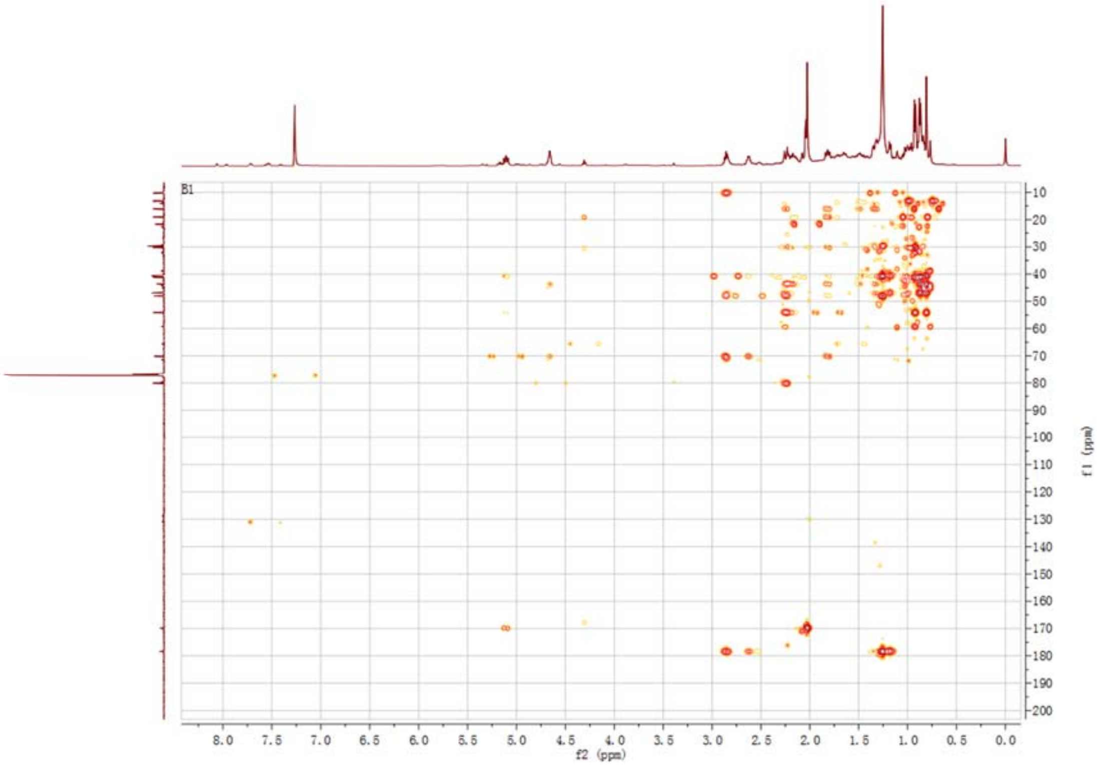

Figure S6. HMBC spectrum of compound **1.**

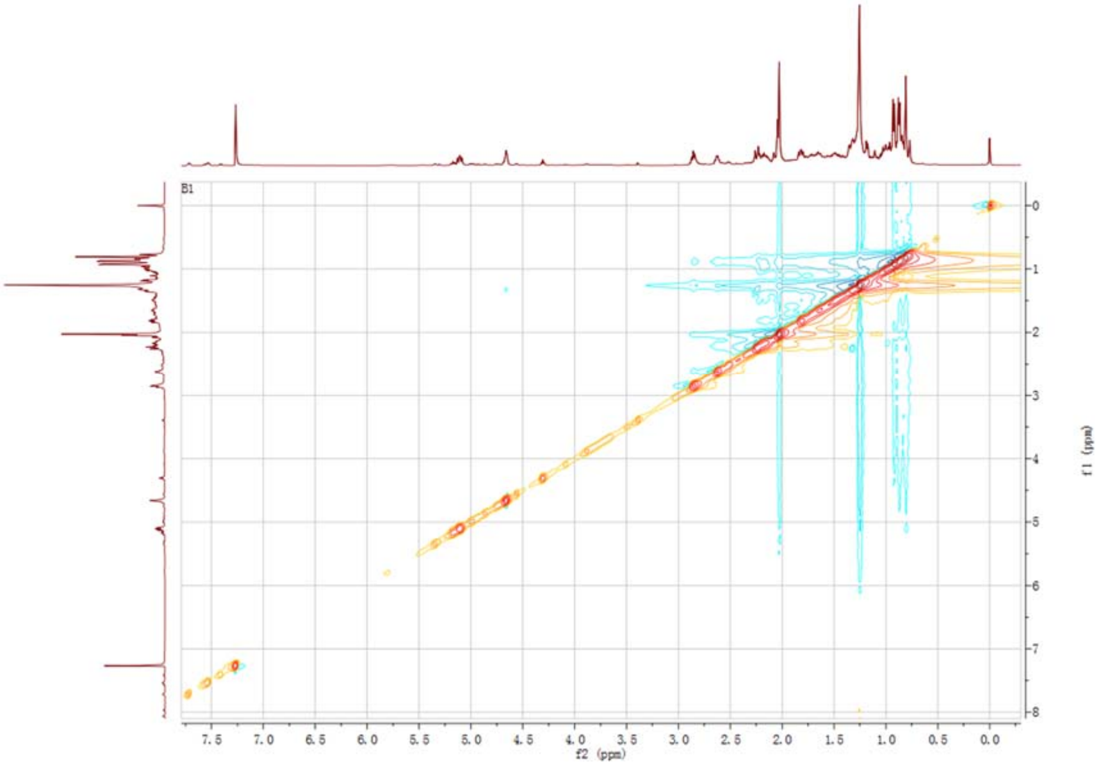

Figure S7. NOESY spectrum of compound **1.**

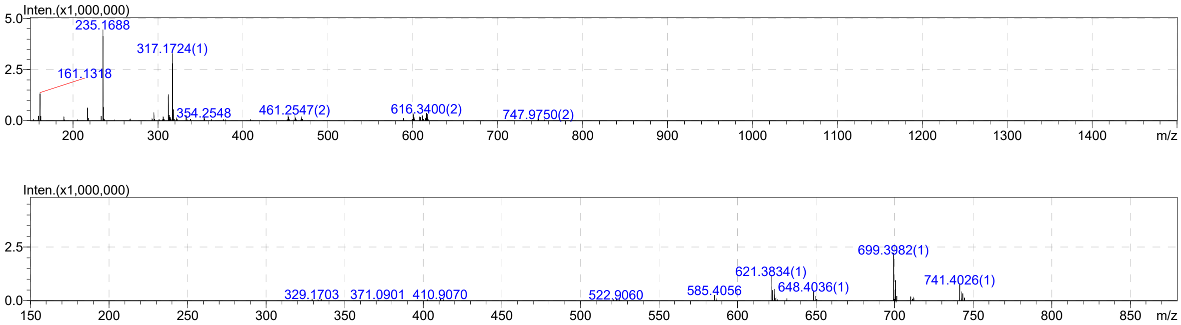

Figure S8. HRESIMS of compound **1.**

S9. The Method of NO Production

NO production was quantified by measuring nitrite accumulation in the culture medium using the Griess reaction. RAW264.7 macrophages were cultured in 96-well plates at a density of 5.5 × 10^4 cells/well for 24 h, and then treated with compound 1 at various concentrations with or without LPS (1μg/ml). Wells with no test compound received only LPS served as controls; Wells with neither any test compound nor LPS served as blank controls. After 24 h stimulation, supernatants were obtained and mixed with an equal volume of Griess reagent (Beyotime Biotechnology, China). NaNO2 was used to generate a standard curve, and nitrite production was determined by measuring optical density at 540 nm using a microplate reader.

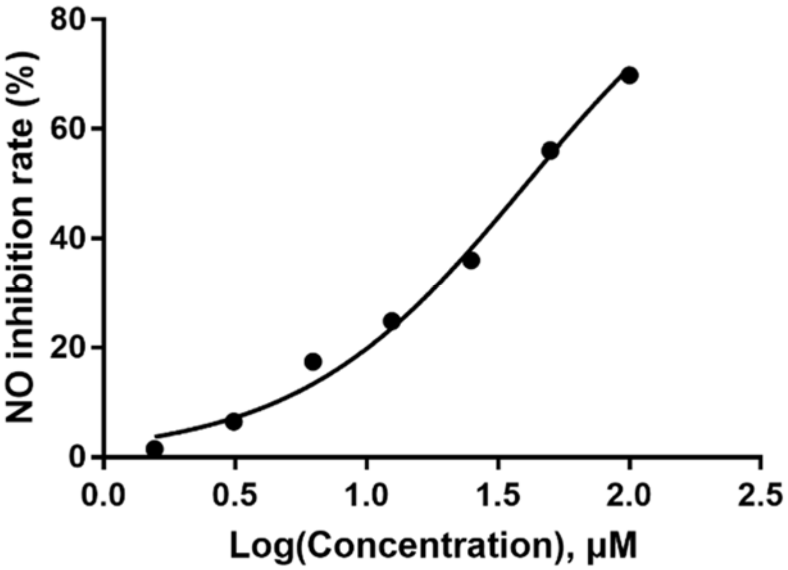

Figure S9. Dose-effect curve of compound **1** on LPS-induced NO inhibition.
